# The cross-sectional relationship between vitamin C and high-sensitivity C-reactive protein levels: insights from NHANES database

**DOI:** 10.3389/fnut.2023.1290749

**Published:** 2023-11-10

**Authors:** Ning Ding, Zhao Zeng, Ju Luo, Keng Li

**Affiliations:** ^1^Department of Emergency Medicine, The Affiliated Changsha Central Hospital, Hengyang Medical School, University of South China, Changsha, China; ^2^Department of Geriatrics, The Affiliated Changsha Central Hospital, Hengyang Medical School, University of South China, Changsha, China

**Keywords:** vitamin C, ascorbic acid, high-sensitivity C-reactive protein, inflammation, NHANES

## Abstract

**Background:**

Ascorbic acid or vitamin C has antioxidant and anti-inflammatory properties that may impact markers of inflammation like C-reactive protein (CRP). However, studies specifically on vitamin C and high-sensitivity CRP (hs-CRP) have been scarce.

**Methods:**

We conducted a cross-sectional analysis of the National Health and Nutrition Examination Survey 2017–2018 dataset including 5,380 U.S. adults aged ≥20 years. Multiple regression models examined the relationship between plasma vitamin C and serum hs-CRP while adjusting for potential confounders. Stratified analyses and curve fitting assessed effect modification and nonlinearity.

**Results:**

An inverse association was found between plasma vitamin C and serum hs-CRP overall (β = −0.025, 95% CI: −0.033 to −0.017, *p* < 0.00001) and in subgroups except for the “other Hispanic” subgroup in model II (β = −0.009, 95% CI: (−0.040, 0.023), *p* = 0.5885). The relationship was nonlinear, with the greatest hs-CRP reduction observed up to a plasma vitamin C level of 53.1 μmol/L.

**Conclusion:**

The results showed a non-linear negative correlation between vitamin C levels and hs-CRP in adults. These results suggest vitamin C intake may reduce inflammation and cardiovascular risk, but only up to 53.1 μmol/L plasma vitamin C.

## Introduction

In recent years, there has been a growing interest in exploring the possible influence of nutrition on inflammation and its related markers. Vitamin C, another name for ascorbic acid, is an essential micronutrient that participates in various biological processes. Vitamin C has immuno-enhancing and anti-inflammatory properties (1, 2), improving immune cell activity and trafficking while dampening overactive immune responses (3, 4). Additionally, vitamin C confers multiple benefits, such as facilitating collagen synthesis (5, 6), and modulating epigenetic processes (7, 8) highlighting its significance in maintaining overall health.

C-reactive protein (CRP) is a highly accurate indicator of widespread inflammation generated by the liver as an acute-phase reactant (9, 10). High-sensitivity CRP (hs-CRP) assay can measure lower levels of CRP in the blood and is a reliable predictor of cardiovascular disease risk (11–14). hs-CRP reflects the degree of systemic inflammation caused by various inflammatory stimuli, such as cytokines and oxidative stress (15). hs-CRP can also activate endothelial cells and stimulate the production of adhesion molecules and pro-inflammatory cytokines, which are important in the progression of atherosclerosis (16–18). Elevated hs-CRP levels are linked to an elevated risk of events associated with cardiovascular disease, such as myocardial infarction (19, 20), stroke recurrence (21, 22), and mortality (23, 24).

Previous research has discovered an inverse relationship between vitamin C status and CRP (25, 26). However, investigations specifically focusing on the relationship between vitamin C and hs-CRP, a more accurate diagnostic marker, have been limited.

To address this research gap, we conducted a cross-sectional analysis of data from the 2017–2018 National Health and Nutrition Examination Survey (NHANES) involving a large and diverse sample of US adults. The NHANES is a nationally representative study that collects comprehensive data regarding the health, nutritional status, and lifestyle of individuals in the United States. By rigorously considering various confounding factors and conducting subgroup analyses, our study aimed to develop a more nuanced comprehension of the possible connections between vitamin C and hs-CRP. Through accounting for potential effect modifiers and sources of heterogeneity, we sought to contribute valuable insights into the interplay between vitamin C and inflammation markers, shedding light on its potential implications for overall health and disease prevention.

## Methods

### Study design

**Figure 1:**
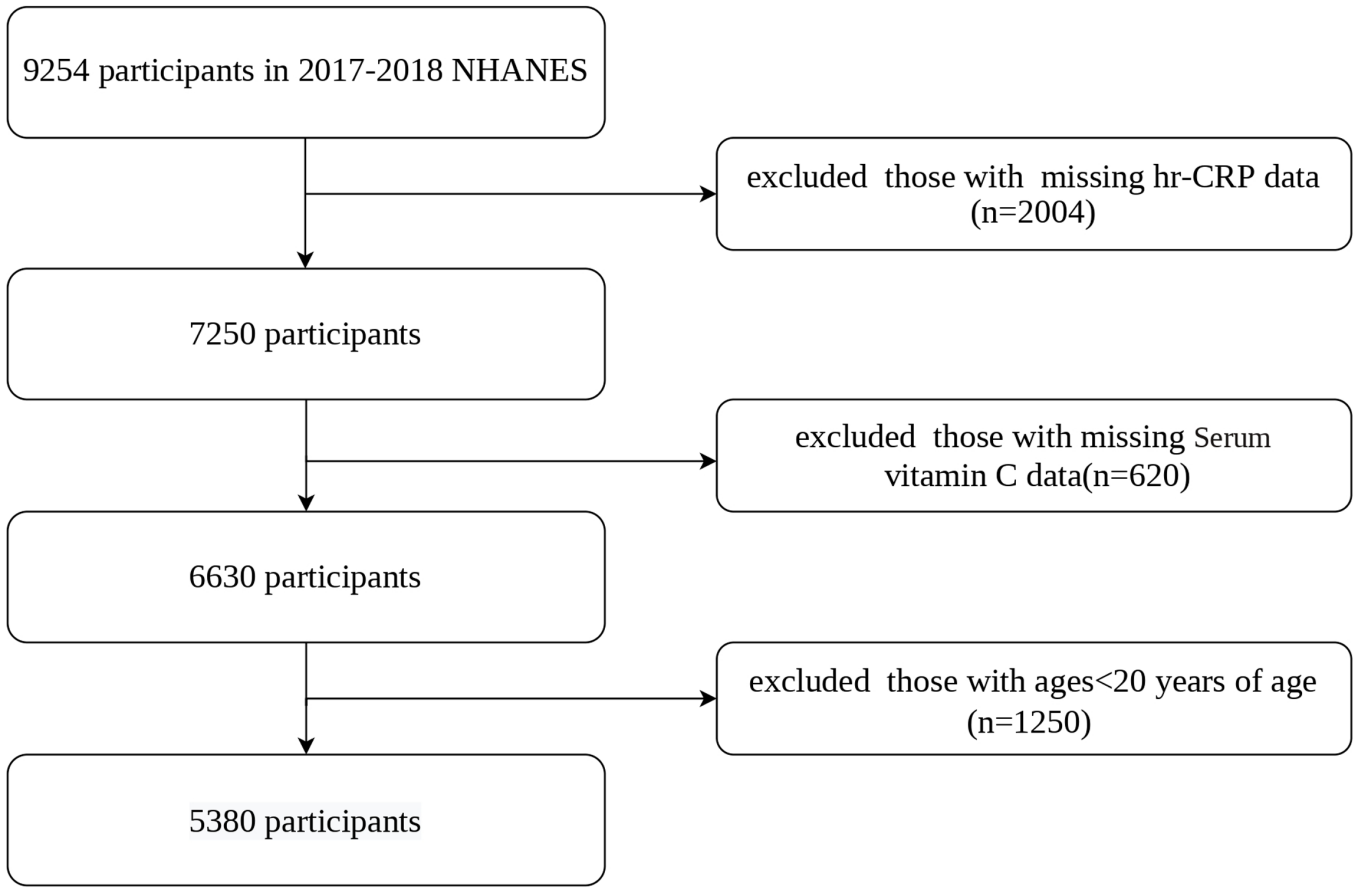
Flowchart of participants selection. NHANES, National Health and Nutrition Examination Survey; hs-CRP, high-sensitivity C-reactive protein.

This cross-sectional research examined NHANES 2017–2018 data. The NCHS Research Ethics Review Board approved the procedure (Protocol #2011–17, Protocol #2018–01). The Centers for Disease Control and Prevention got written and informed permission from all participants. We included participants aged 20 years and older who had complete data for serum vitamin C and hs-CRP levels, 5,380 adults aged ≥20 years were included out of 9,254 initial participants. Figure 1 illustrates the detailed screening process.

### Measurement of serum vitamin C and hs-CRP

Serum vitamin C was obtained and measured using isocratic high-performance liquid chromatography (HPLC) with electrochemical detection at 650 mV. A standard curve of three concentrations (0.025, 0.150, and 0.500 mg/dL) of an external standard was used for quantification. The NHANES reliability assurance and quality control processes satisfied the standards of the Clinical Laboratory Improvement Act of 1988. Serum vitamin C levels were analyzed continuously and categorically according to a prior study (27, 28): deficiency (0–10.99 μmol/L), hypovitaminosis (11–23.99 μmol/L), inadequate (24–49.99 μmol/L), adequate (50–69.99 μmol/L), and saturating (≥70 μmol/L).

Hs-CRP was quantified using a high-sensitivity near-infrared particle immunoassay method in Mobile Examination Centers (CDC-NHANES, 2019). hs-CRP was reported in mg/dL.

### Covariates

This study included a variety of covariates: demographic characteristics (age, sex, race, and household income-to-poverty ratio), chronic diseases (hypertension and diabetes), lifestyle behaviors (physical activity, smoking, and alcohol consumption), examination data (BMI), supplement use, food security and dietary inflammatory index (DII).

The demographic characteristics were categorical variables, such as: sex (male or female); race (Non-Hispanic Black, Other Race – Including Multi-Racial, Non-Hispanic White, Mexican American, or Other Hispanic); and family income-to-poverty ratio (<=1.5, >1.5, <=4.5, >4.5 or Not Recorded).

Chronic illnesses were evaluated using self-reported medical histories. Hypertension has been characterized as having any or all of the following criteria: (1) current usage of antihypertensive medication; (2) previous diagnosis of hypertension by a physician; (3) an average systolic blood pressure of 140 mmHg or higher based on four measurements; or (4) a diastolic blood pressure of 90 mmHg or higher. Diabetes was categorized as “yes”, “no”, “borderline”, and “Not Recorded” based on the following criteria: (1) doctor’s diagnosis of diabetes; (2) current use of hypoglycemic medication; (3) fasting blood glucose of 7.0 mmoL/L or higher; (4) glycated hemoglobin of 6.5% or higher; (5) two-hour oral glucose tolerance test blood glucose of 11.1 mmoL/L or higher; and (6) random blood glucose of 11.1 mmoL/L or higher. Physical activity was classified as “yes”, “no”, and “Not Recorded” based on the World Health Organization guidelines of more than 599 MET minutes/week. Smoking status groups were “never smokers” (less than 100 lifetime cigarettes), “former smokers”, “current smokers”, and “Not Recorded”. Alcohol consumption categories were drinkers, non-drinkers, and Not Recorded based on the past 12 month intake frequency. Food security was evaluated based on the answer to the question, “Are you worried you will run out of food?” It was categorized as “yes”, “no”, or “not recorded”. Supplement use was examined based on the reply to the question. “Any Dietary Supplements Taken?” It was recorded as “Yes”, “No”, or “Not Recorded”. Weight and height measurements were used to determine the body mass index (BMI).

The DII assesses the inflammatory impacts of dietary consumption based on 45 specific nutrients. Each component’s score is determined by calculating the deviation from the global daily mean intake, dividing it by the standard deviation, and converting the resulting Z score to a percentile score. After doubling the percentile score and subtracting it by 1 for a symmetrical distribution, the percentile value is multiplied by the corresponding “overall inflammation effect score” for that nutrient. Summing up all individual DII scores yields the “overall DII score” for each participant. Our study computed the DII score using 28 nutrients, including alcohol, vitamin B12/B6, β-carotene, caffeine, carbohydrate, cholesterol, energy, total fat, fiber, folic acid, iron (Fe), magnesium (Mg), monounsaturated fatty acids (MUFA), niacin, n-3 fatty acids, n-6 fatty acids, protein, polyunsaturated fatty acids (PUFA), riboflavin, saturated fat, selenium (Se), thiamin, vitamins A/C/D/E, and zinc (Zn).

## Statistical analysis

Weighted data from NHANES 2017–2018 were produced in compliance with analytical criteria. Vitamin C was assessed using both quintiles and continuous variables. Linear regression models were applied to investigate the connection between vitamin C and hs-CRP. Three different models were built: crude Model was unadjusted; Model I was adjusted for sex, age, and race; and Model II was adjusted for all covariates (sex, age, race, poverty income ratio level, body mass index, food security, supplement use, hypertension, diabetes, physical activity, smoking status, alcohol consumption and dietary inflammatory index). Stratified analysis and smooth curve fitting have been applied to further investigate the association between vitamin C and hs-CRP. To determine the independent impacts of Vitamin C on hs-CRP, univariate and stratified analyses were performed. Continuous data are shown as means and standard errors, and categorical variables are shown as percentages. Effect estimates are reported as β coefficients with corresponding 95% confidence intervals. A 0.05 or lower *p*-value was considered statistically significant. Missing values for BMI were replaced with the mean value, and missing categorical data were analyzed as a single group in the analysis. The entire data analysis had been utilized using R (version 4.2.0; http://www.r-project.org) and Empower Stats (http://www.empowerstats.com; X&Y Solutions Inc.).

## Result

We analyzed the data of 5,380 participants from the NHANES 2017–2018. Table 1 presents the weighted characteristics that distinguish the study population according to plasma vitamin C quintiles (range < 11 to > = 70 μmol/L). All the variables showed significant linear trends across the vitamin C quintiles (*p* < 0.05). The prevalence of hypertension decreased from 45.6% in the lowest vitamin C quintile (<11 μmol/L) to 35.6% in the highest vitamin C quintile (> = 70 μmol/L) (*P* for trend <0.0001). The mean BMI also decreased from 30.6 ± 8.4 kg/m2 in the lowest vitamin C quintile to 26.7 ± 7.0 kg/m2 in the highest vitamin C quintile (*P* for trend <0.0001). The proportion of females increased from 46.5% in the lowest vitamin C quintile to 67.7% in the highest vitamin C quintile (*P* for trend <0.0001). Supplement use increased from 25.0% in the lowest vitamin C quintile to 77.8% in the highest vitamin C quintile (*P* for trend <0.0001). Never smokers increased from 34.5% in the lowest vitamin C quintile to 57.5% in the highest vitamin C quintile (*P* for trend <0.0001). We stratified vitamin C and hs-CRP data by vitamin C levels, and Supplementary Table S1 shows a univariate analysis of the relationship between variables and hs-CRP.

**Table 1:** Weighted characteristics of the study population based on vitamin C quintiles.

Vitamin C categories (μmol/L)	<11	> = 11, <24	> = 24, <50	> = 50, <70	> = 70	*p* value
Age mean + SD (years)	48.812 ± 16.894	45.144 ± 16.664	46.334 ± 17.415	45.746 ± 18.489	47.106 ± 22.721	0.01742
BMI mean + SD (kg/m^2^)	30.599 ± 8.414	32.072 ± 8.303	30.524 ± 7.505	28.642 ± 6.688	26.731 ± 6.995	<0.00001
hs-CRP mean + SD (mg/L)	5.808 ± 10.424	5.042 ± 9.525	4.491 ± 9.643	3.046 ± 5.030	2.776 ± 4.853	<0.00001
DII	2.470 ± 1.593	2.148 ± 1.507	1.591 ± 1.752	1.167 ± 1.850	1.262 ± 1.852	<0.00001
Sex (%)						<0.00001
Female	46.503	41.351	46.041	48.019	67.731	
Male	53.497	58.649	53.959	51.981	32.269	
Race/ethnicity (%)						<0.00001
Non-Hispanic Black	9.342	10.889	13.634	10.528	8.413	
Other Race- including multi-racial	8.991	8.924	12.231	11.309	8.794	
Non-Hispanic White	72.203	69.231	52.828	60.502	68.352	
Mexican American	5.197	6.804	12.211	10.279	7.931	
Other Hispanic	4.266	4.152	9.096	7.382	6.510	
Supplement use (%)						<0.00001
Yes	24.981	34.273	51.871	60.009	77.837	
No	75.019	65.661	48.079	39.964	22.130	
Not Recorded	0.000	0.066	0.050	0.027	0.032	
Food insecure (%)						<0.00001
No	63.586	70.348	70.117	75.949	75.619	
Yes	33.960	25.661	25.370	21.002	20.387	
Not recorded	2.454	3.991	4.512	3.049	3.994	
Physical activity (%)						<0.00001
No	10.947	10.630	12.151	9.193	10.628	
Yes	60.575	63.582	65.066	68.905	58.345	
Not recorded	28.478	25.787	22.783	21.902	31.026	
Family PIR (%)						<0.00001
<=1.5	32.783	25.868	23.784	19.385	18.478	
>1.5, <=4.5	36.847	33.032	37.380	38.408	40.599	
>4.5	19.933	30.521	27.402	32.412	31.004	
Not recorded	10.437	10.579	11.435	9.795	9.919	
Alcohol consumption (%)						<0.00001
No drinking	8.801	4.663	6.371	6.065	7.201	
Drinking	63.286	73.297	72.774	70.758	60.548	
Not recorded	27.913	22.040	20.856	23.177	32.252	
Smoking status (%)						<0.00001
Former	21.791	26.263	25.897	23.027	22.141	
Never	34.510	47.847	55.223	58.366	57.518	
Now	43.551	25.075	16.910	14.010	7.493	
Not recorded	0.149	0.815	1.970	4.597	12.848	
Diabetes (%)						<0.00001
No	77.475	69.248	71.873	79.204	81.872	
Borderline	5.314	9.893	8.929	6.065	5.880	
Yes	17.211	19.226	18.471	13.793	11.333	
Not recorded	0.000	1.634	0.727	0.938	0.914	
Hypertension (%)						<0.00001
Yes	45.627	41.374	40.903	34.592	35.585	
No	54.373	58.445	58.250	63.701	57.672	
Not recorded	0.000	0.180	0.846	1.706	6.743	

Mean ± SD for continuous variables: *p*-value was calculated by weighted linear regression model. % for categorical variables: *p*-value was calculated by weighted chi-square test. BMI, body mass index; PIR, household income-to-poverty ratio; hs-CRP, high-sensitivity C-reactive protein; DII, dietary inflammatory index.

In Table 2, we carried out a multivariate regression analysis to analyze the relationship between vitamin C (μmol/L) and hs-CRP (mg/L) levels while adjusting for various covariates. The Crude Model revealed a significant negative relationship between vitamin C and hs-CRP (β = −0.034, 95% CI: −0.041 to −0.027, *p* < 0.00001). After adjusting for sex, age, and race/ethnicity in Model I, the negative association remained highly significant (β = −0.038, 95% CI: −0.045 to −0.031, *p* < 0.00001). Further adjustments for additional covariates in Model II also showed a strong negative association (β = −0.024, 95% CI: −0.032 to −0.016, *p* < 0.00001). Furthermore, the categorical analysis revealed that increased vitamin C levels resulted in gradually reduced hs-CRP levels, with the strongest effect observed in the highest category (> = 70 μmol/L, β = −2.383 to −3.032).

**Table 2:** Relationship between vitamin C (μmol/L) and hs-CRP (mg/L) levels in different models.

Variable	Crude model	Model I	Model II
	β (95%CI) *p*-value	β (95%CI) *p*-value	β (95%CI) *p*-value
Vitamin C (μmol/L)	−0.034 (−0.041, −0.027) <0.00001	−0.038 (−0.045, −0.031) <0.00001	−0.024 (−0.032, −0.016) <0.00001
Vitamin C categories (μmol/L)			
<11	Reference	Reference	Reference
> = 11, <24	−0.766 (−1.750, 0.219) 0.12752	−0.619 (−1.598, 0.360) 0.21510	−0.971 (−1.928, −0.015) 0.04663
> = 24, <50	−1.317 (−2.212, −0.422) 0.00395	−1.316 (−2.209, −0.422) 0.00391	−1.215 (−2.105, −0.325) 0.00747
> = 50, <70	−2.762 (−3.651, −1.873) <0.00001	−2.742 (−3.626, −1.857) <0.00001	−2.129 (−3.027, −1.231) <0.00001
> = 70	−3.032 (−3.935, −2.129) <0.00001	−3.300 (−4.202, −2.399) <0.00001	−2.383 (−3.327, −1.440) <0.00001
*P* for trend	<0.001	<0.001	<0.001

CI, confidence interval; hs-CRP, high-sensitivity C-reactive protein. Crude Model no covariates were adjusted. Model I adjusted for sex, age, and race/ethnicity. Model II adjusted for sex, age, race/ethnicity, family poverty income ratio level, body mass index, hypertension, diabetes, physical activity, food insecure, supplement use, body mass index, smoking status and alcohol consumption, dietary inflammatory index.

Additionally, Table 3 displays the correlation between vitamin C levels (μmol/L) and hs-CRP levels (mg/L) in various subgroups stratified by age, sex, race/ethnicity, smoking status, and alcohol consumption. The findings demonstrate a negative connection between vitamin C and hs-CRP in all subgroups and models, except for the “other Hispanic” subgroup in model II (β = −0.007, 95% CI: (−0.038, 0.024), *p* = 0.6429).The strength of the negative correlation varied among subgroups, with the “other race – including multi-racial” subgroup showing the strongest correlation (β = −00.051, 95% CI: (−0.074, −0.029), *p* < 0.0001 in model II), and the “former smoker” subgroup showing the weakest correlation (β = −0.013, 95% CI: (−0.028, 0.002), *p* = 0.0791 in model II).

**Table 3:** Stratified analysis of the correlation between vitamin C (μmol/L) and hs-CRP (mg/L).

	Crude Model	Model I	Model II
	β (95%CI) *p*-value	β (95%CI) *p*-value	β (95%CI) *p*-value
Subgroup analysis stratified by age			
<60	−0.035 (−0.043, −0.026) <0.0001	−0.0369 (−0.047, −0.030) <0.0001	−0.020 (−0.029, −0.010) <0.0001
> = 60	−0.034 (−0.046, −0.022) <0.0001	−0.038 (−0.050, −0.027) <0.0001	−0.029 (−0.041, −0.017) <0.0001
Subgroup analysis stratified by sex			
Male	−0.035 (−0.046, −0.024) <0.0001	−0.035 (−0.046, −0.024) <0.0001	−0.025 (−0.037, −0.014) <0.0001
Female	−0.040 (−0.050, −0.031) <0.0001	−0.041 (−0.050, −0.031) <0.0001	−0.023 (−0.033, −0.013) <0.0001
Subgroup analysis stratified by race/ethnicity			
Non-Hispanic Black	−0.042 (−0.066, −0.018) 0.0005	−0.042 (−0.066, −0.018) 0.0006	−0.027 (−0.051, −0.004) 0.0235
Other race – including multi-racial	−0.062 (−0.085, −0.039) <0.0001	−0.067 (−0.090, −0.045) <0.0001	−0.051 (−0.074, −0.029) <0.0001
Non-Hispanic White	−0.029 (−0.037, −0.021) <0.0001	−0.035 (−0.043, −0.026) <0.0001	−0.021 (−0.030, −0.012) <0.0001
Mexican American	−0.041 (−0.069, −0.014) 0.0035	−0.040 (−0.068, −0.013) 0.0040	−0.025 (−0.052, 0.002) 0.0741
Other Hispanic	−0.020 (−0.052, 0.012) 0.2177	−0.026 (−0.057, 0.006) 0.1172	−0.007 (−0.038, 0.024) 0.6429
Subgroup analysis stratified by smoking status			
Former	−0.016 (−0.031, −0.001) 0.0360	−0.023 (−0.038, −0.008) 0.0029	−0.013 (−0.028, 0.002) 0.0791
Never	−0.036 (−0.046, −0.026) <0.0001	−0.043 (−0.053, −0.033) <0.0001	0.027 (−0.036, −0.017) <0.0001
Now	−0.039 (−0.059, −0.020) <0.0001	−0.039 (−0.059, −0.020) <0.0001	−0.029 (−0.048, −0.010) 0.0024
Not recorded	−0.029 (−0.065, 0.007) 0.1091	−0.031 (−0.067, 0.005) 0.0880	−0.028 (−0.063, 0.007) 0.1196
Subgroup analysis stratified by alcohol consumption			
No drinking	−0.046 (−0.070, −0.021) 0.0003	−0.053 (−0.077, −0.028) <0.0001	−0.034 (−0.058, −0.010) 0.0059
Drinking	−0.025 (−0.034, −0.016) <0.0001	−0.032 (−0.041, −0.023) <0.0001	−0.016 (−0.026, −0.006) 0.0011
Not recorded	−0.052 (−0.065, −0.040) <0.0001	−0.050 (−0.063, −0.037) <0.0001	−0.035 (−0.049, −0.021) <0.0001

CI, confidence interval; hs-CRP, high-sensitivity C-reactive protein. Crude Model no covariates were adjusted. Model I adjusted for sex, age, and race/ethnicity except the subgroup variable. Model II adjusted for sex, age, race/ethnicity, family poverty income ratio level, body mass index, hypertension, diabetes, physical activity, food insecure, supplement use, body mass index, smoking status and alcohol consumption, dietary inflammatory index except the subgroup variable.

**Figure 2:**
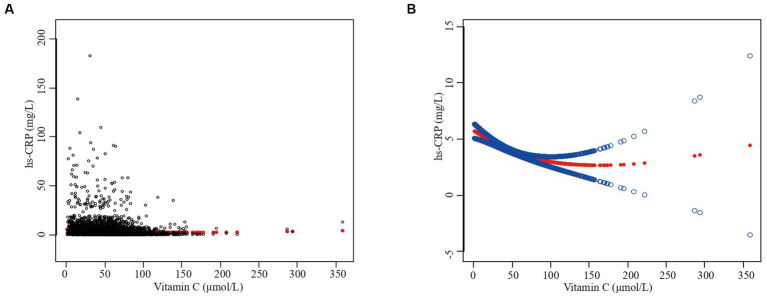
The association between vitamin C and high-sensitivity C-reactive protein. **(A)** Each black point represents a sample. **(B)** Solid redline represents the smooth curve fit between variables. Blue bands represent the 95% of confidence interval from the fit. Sex, age, race/ethnicity, family poverty income ratio level, body mass index, hypertension, diabetes, physical activity, food insecure, supplement use, body mass index, smoking status, dietary inflammatory index and alcohol consumption were adjusted.

**Figure 3:**
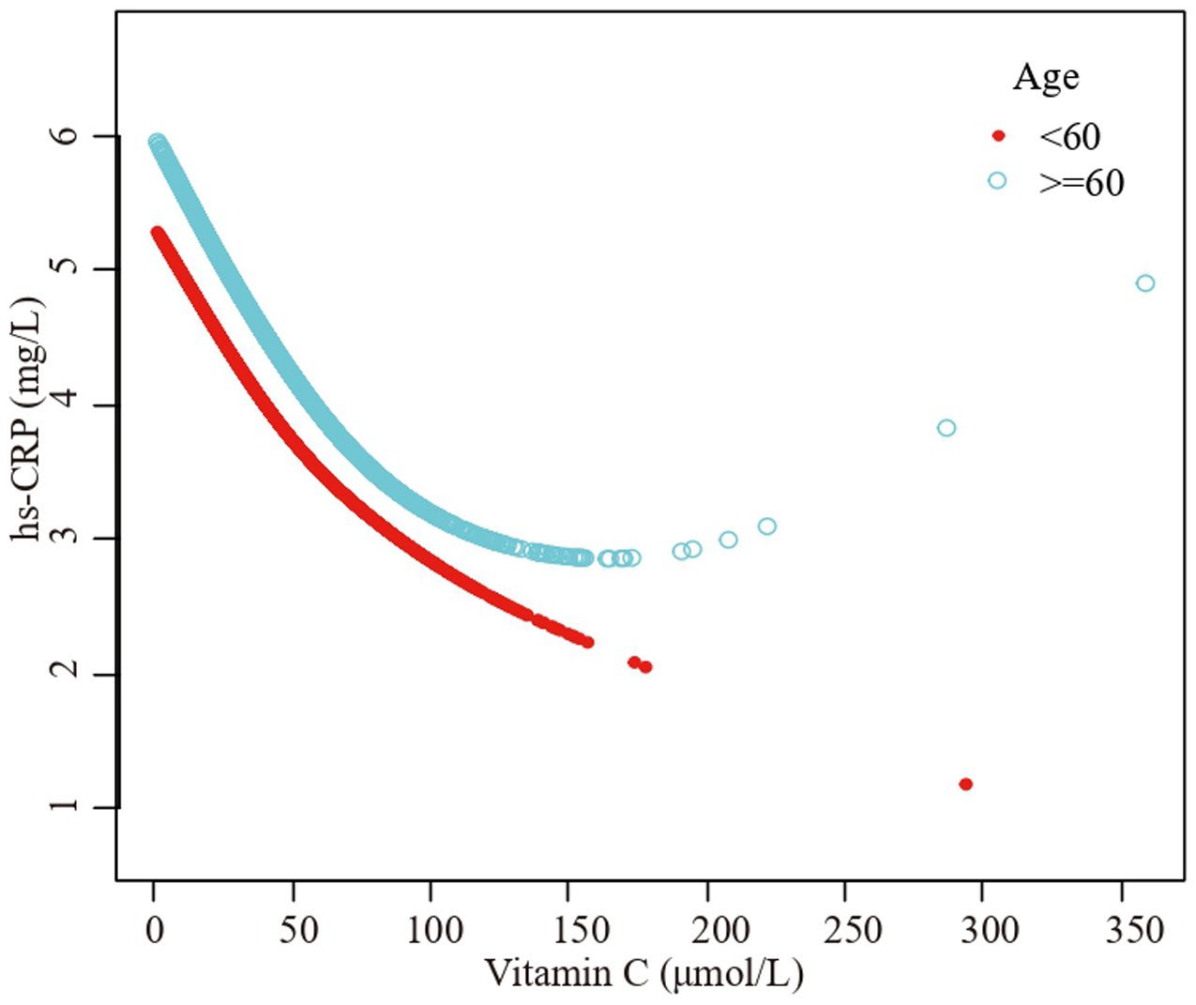
The association vitamin C and high-sensitivity C-reactive protein stratified by age. Sex, race/ethnicity, family poverty income ratio level, body mass index, hypertension, diabetes, physical activity, food insecure, supplement use, body mass index, smoking status, dietary inflammatory index and alcohol consumption were adjusted.

**Figure 4:**
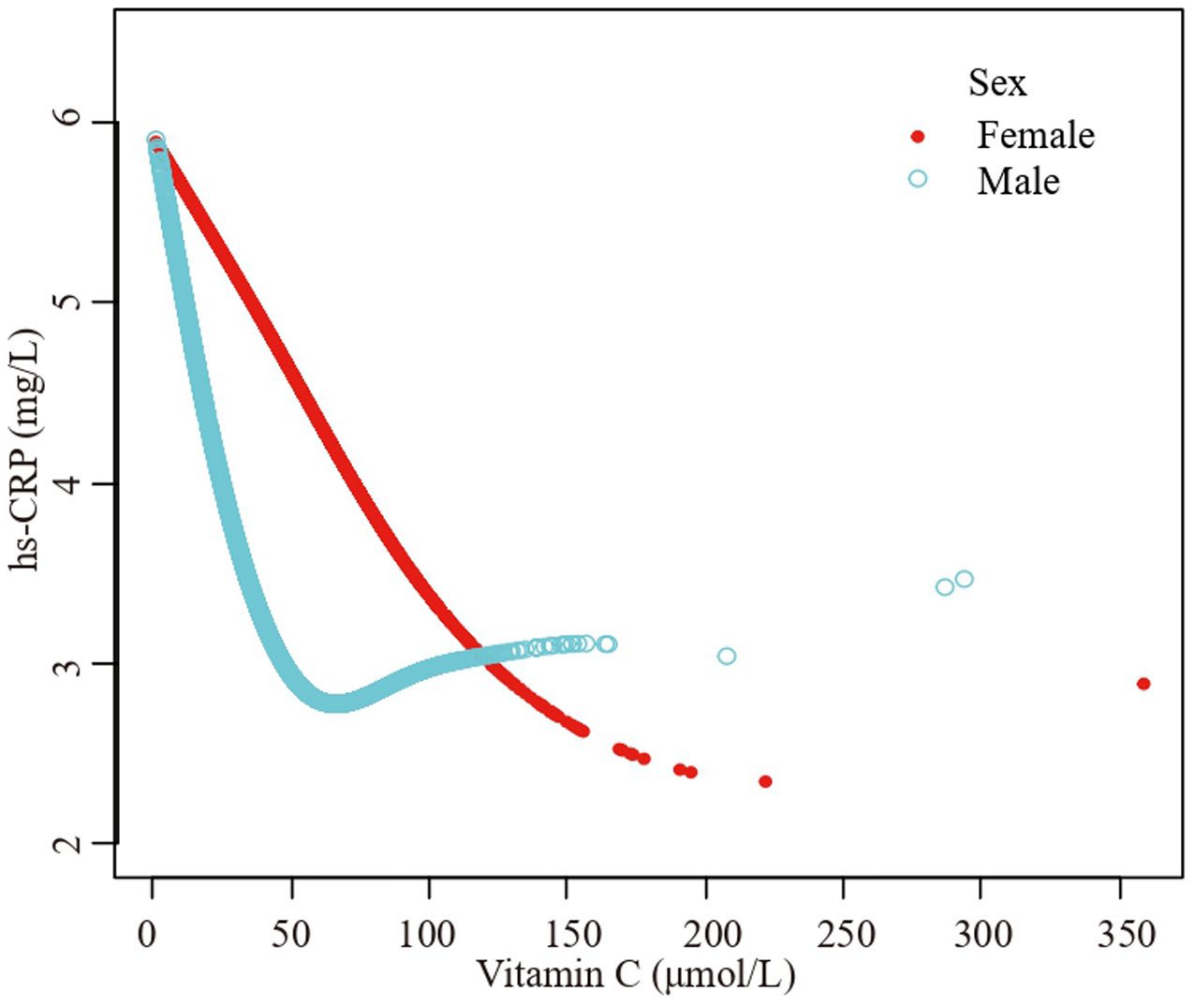
The association vitamin C and high-sensitivity C-reactive protein stratified by sex. Age, race/ethnicity, family poverty income ratio level, body mass index, hypertension, diabetes, physical activity, food insecure, supplement use, body mass index, smoking status, dietary inflammatory index and alcohol consumption were adjusted.

**Figure 5:**
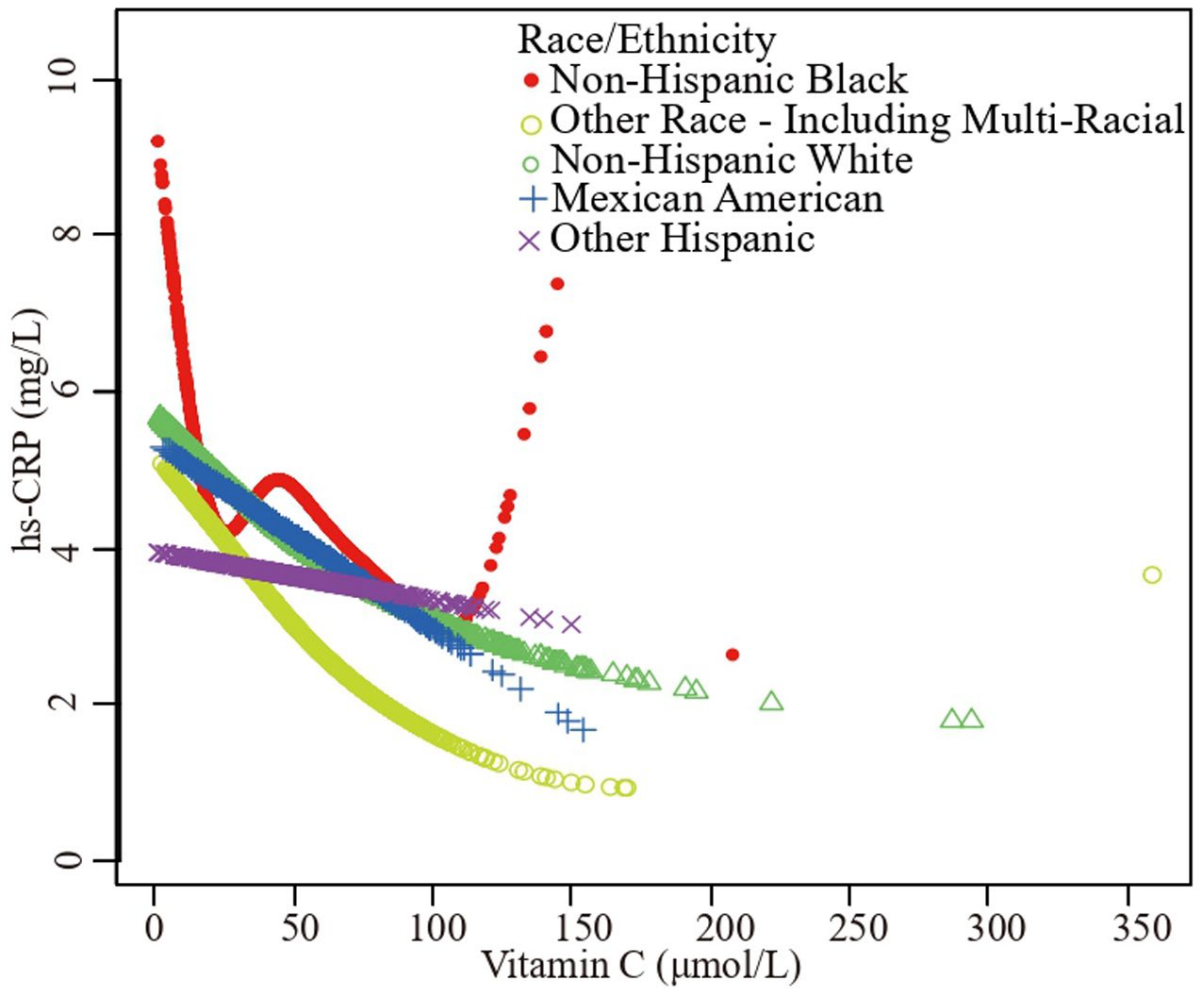
The association vitamin C and high-sensitivity C-reactive protein stratified by race/ethnicity. Age, sex, family poverty income ratio level, body mass index, hypertension, diabetes, physical activity, food insecure, supplement use, body mass index, smoking status, dietary inflammatory index and alcohol consumption were adjusted.

**Figure 6:**
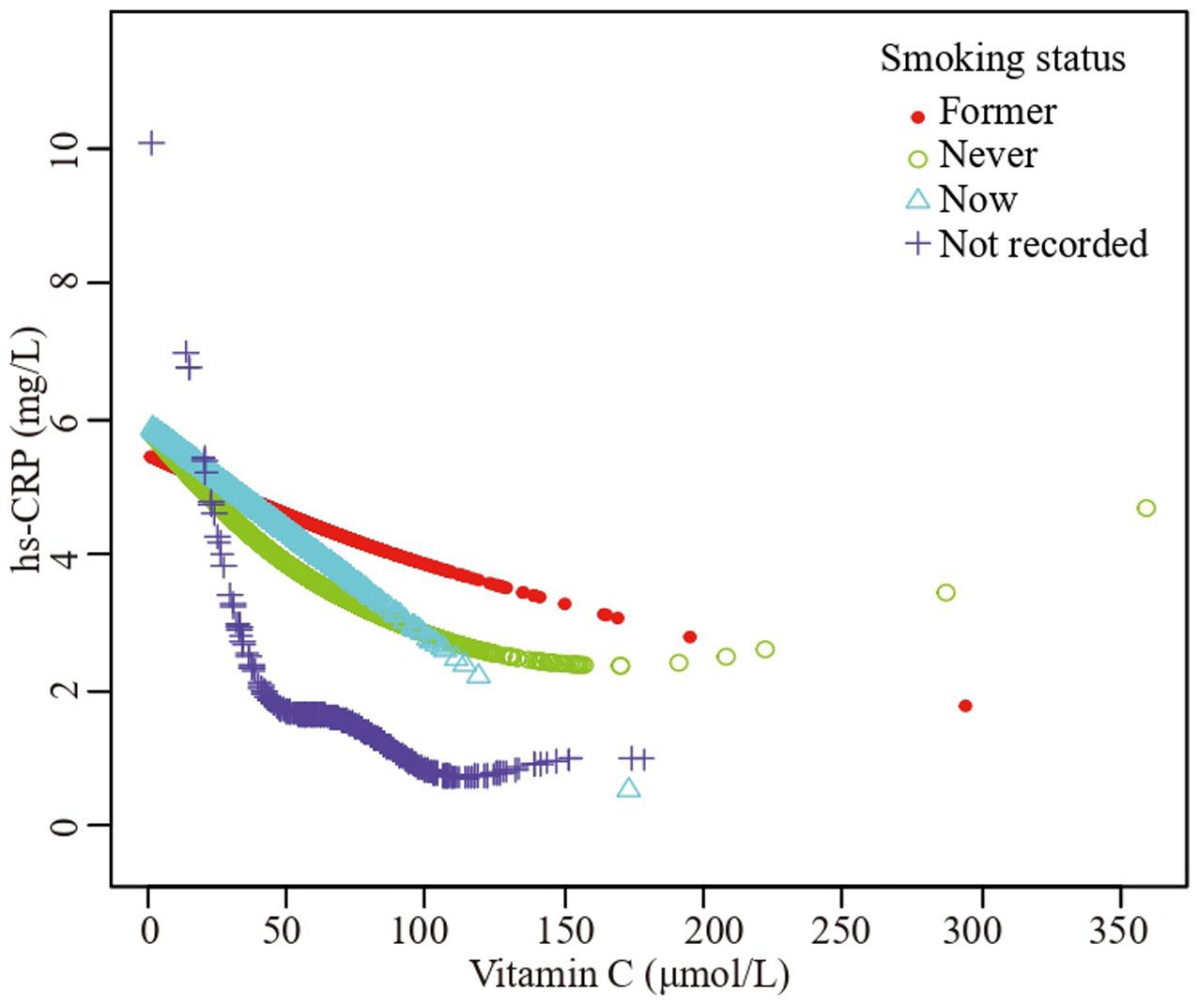
The association vitamin C and high-sensitivity C-reactive protein stratified by smoking status. Sex, age, race/ethnicity, family poverty income ratio level, body mass index, hypertension, diabetes, physical activity, food insecure, supplement use, body mass index, dietary inflammatory index and alcohol consumption were adjusted.

**Figure 7:**
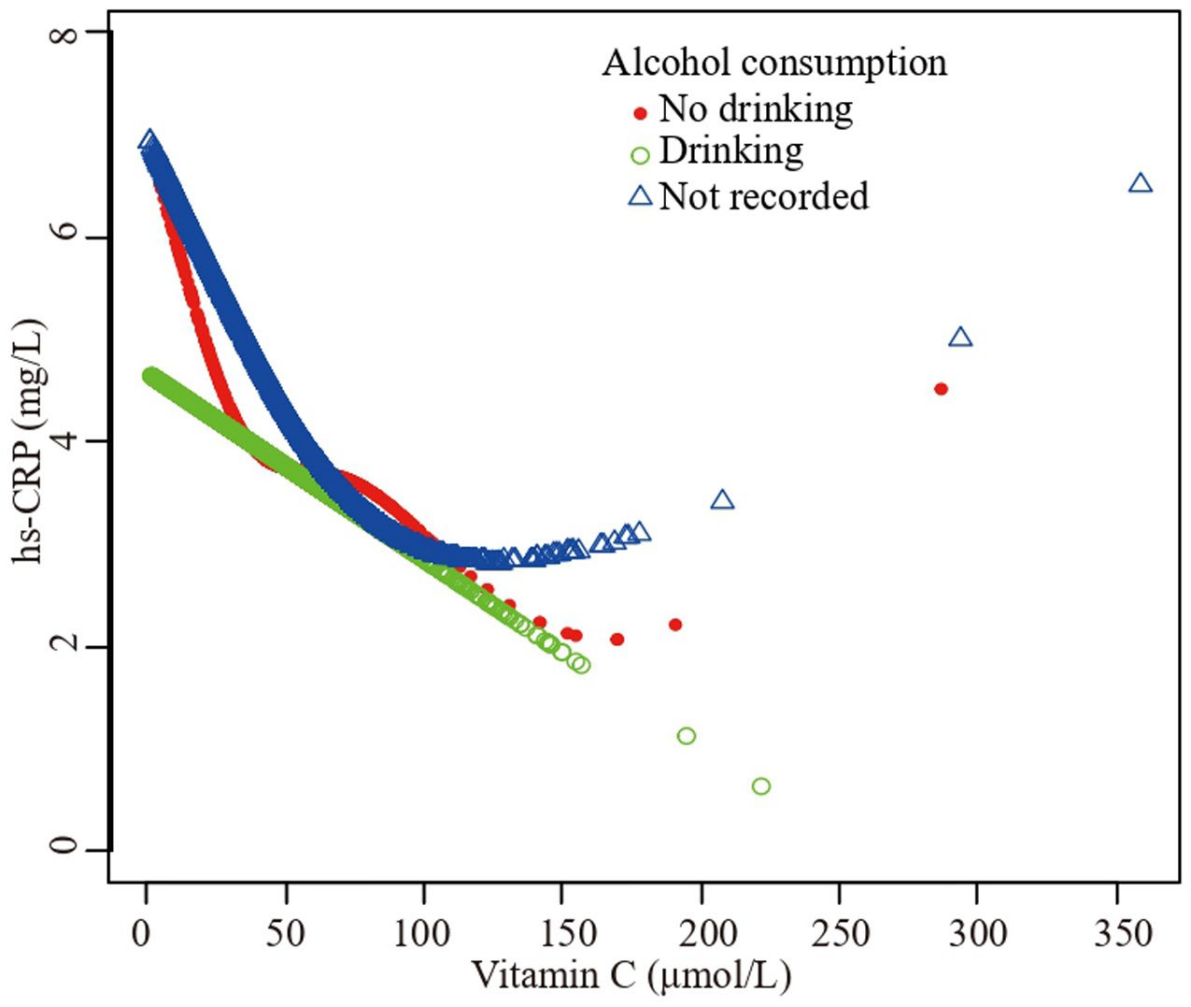
The association vitamin C and high-sensitivity C-reactive protein stratified by alcohol consumption. Sex, age, race/ethnicity, family poverty income ratio level, body mass index, hypertension, diabetes, physical activity, food insecure, supplement use, body mass index, dietary inflammatory index and smoking status were adjusted.

Furthermore, we employed a smooth curve fitting approach to investigate the potential nonlinear connection between vitamin C and hs-CRP, leading to consistent results, as depicted in Figures 2–7. In addition, we ran a log-likelihood ratio test and compared the one-line linear regression model to the two-segment regression model. A two-step recursive technique was used to determine the infection point, *K* = 53.1. The results showed an optimal level of vitamin C, approximately 53.1 μmol/L, which was associated with the most significant reduction in hs-CRP levels. The results are shown in Table 4.

**Table 4:** Threshold effect analysis of vitamin C (μmol/L) and hs-CRP (mg/L) using piece-wise linear regression.

Models	β (95%CI)	*p* value
Model I		
One line effect	−0.017 (−0.034, 0.000)	0.0511
Model II		
Infection point (*K*)	53.1	
Vitamin C < 53.1 (μmol/L)	−0.091 (−0.139, −0.043)	0.0002
Vitamin C > 53.1 (μmol/L)	−0.006 (−0.024, 0.012)	0.5094
*P* for log-likelihood ratio test		0.001

Model I, one-line linear regression; Model II, two-segment regression. β was the effect size and the 95% CI indicated the confidence interval adjusted for sex, age, race/ethnicity, family poverty income ratio level, body mass index, hypertension, diabetes, physical activity, food insecure, supplement use, body mass index, smoking status and alcohol consumption, dietary inflammatory index.

## Discussion

In this cross-sectional investigation, we analyzed the relationship between vitamin C and hs-CRP using the NHANES 2017–2018 database. Multiple regression models, stratified analysis, and smooth curve fitting were applied to investigate the linear and nonlinear association between vitamin C and hs-CRP. The results revealed a non-linear negative correlation between vitamin C levels and hs-CRP in adults. A significant decrease of hs-CRP was observed until vitamin C reached 53.1 μmol/L.

Our findings are consistent with prior research that found a negative relationship between vitamin C and hs-CRP in different populations and settings. Lin et al. (29) found a high prevalence of low plasma vitamin C in a subtropical region, which was linked to high hs-CRP levels. Several studies found lower plasma vitamin C and higher hs-CRP levels in patients with chronic diseases such as respiratory diseases (30), hypertension, diabetes, and obesity (31) compared to healthy controls or placebo groups. Other studies reported that vitamin C supplementation or intake significantly reduced CRP levels in various subgroups such as males, non-smokers, healthy subjects, younger people, and at doses <500 mg/day (32). Some studies also found an inverse connection between CRP and vitamin C levels in special populations such as pregnant women with depression and anxiety (33) and patients with septic cardiomyopathy (34).

The inverse connection between vitamin C and hs-CRP is thought to be mediated by several potential mechanisms involving antioxidant and anti-inflammatory effects of vitamin C. First, as an antioxidant, vitamin C is essential for scavenging reactive oxygen species and decreasing oxidative stress, which in turn helps to reduce the inflammatory cascade (35–37). Second, vitamin C is believed to possess anti-inflammatory properties, potentially through its ability to enhance immune cell function and preserve endothelial integrity (38, 39). Third, vitamin C may contribute to the reduction of inflammation by inhibiting the apoptosis of endothelial cells and monocytes (38), both of which are associated with the pathogenesis of atherosclerosis (40).

However, research on the relationship between vitamin C and CRP levels has also shown inconsistent results. Several studies found a non-significant difference in CRP levels between vitamin C and placebo teams among sepsis patients (41, 42). A meta-analysis found no overall significant influence of vitamin C and E co-supplementation on plasma CRP levels, with varied results in different populations (43). Another study reported that 100% orange juice intake for 4 weeks significantly reduced interleukin-6 levels but had no significant effect on CRP concentrations (44). The inconsistent findings regarding vitamin C and CRP across studies may be due to differences in several factors such as study design, participants, vitamin C doses, intervention duration, and confounding factors.

Besides these factors, our research has several limitations that should be mentioned. First, the cross-sectional method makes it impossible to demonstrate causality throughout vitamin C and hs-CRP. Second, we used the NHANES database, which only represents the adult population aged 20 years old and above in the United States. Therefore, we cannot generalize our results to other age groups or populations outside of the United States. Third, the lack of detailed reporting on the standardized timing of blood collection may have influenced the measured biomarkers. Further investigation is necessary to validate and extend our findings to broader demographics and explore the underlying mechanisms between vitamin C and hs-CRP using more robust study designs. Moreover, there could be additional combined factors that were not thoroughly considered, which could impact the correlation between vitamin C and hs-CRP. Further investigation is required to validate and broaden our findings to broader demographics and explore the underlying mechanisms between vitamin C and hs-CRP using more robust study designs.

Despite these limitations, our study also has some strengths and innovations that should be highlighted. One of the strengths is that we utilized a weighted nationally representative sample comprising a diverse multiracial population, ensuring that our findings are highly representative of the entire population. Another innovation is that we added new insights by exploring the nonlinear relationship between vitamin C and hs-CRP through the implementation of smooth curve fitting. Intriguingly, our results revealed an optimal level of vitamin C, approximately 53.1 μmol/L, which was associated with the most significant reduction in hs-CRP levels. This observation suggests that a higher intake of vitamin C might be beneficial in mitigating inflammation and reducing cardiovascular risk, but only up to a certain threshold. We recommend following the current nutritional guidelines and obtaining adequate vitamin C from food sources to determine the optimal vitamin C intake, rather than relying excessively on supplements, before further research is conducted. These findings have significant implications for both public health and therapeutic practice.

## Conclusion

Based on our study, we demonstrated a negative and nonlinear relationship between plasma vitamin C and hs-CRP in an overall representative sample of US adults. Our findings suggest that optimal vitamin C intake may have anti-inflammatory and cardioprotective effects, but further research is warranted to establish the causal relationship and the underlying mechanisms. We recommend increasing vitamin C intake to optimal levels for reducing inflammation and cardiovascular risk, but further research is warranted to establish the causal relationship and the underlying mechanisms.

## Data availability statement

The raw data supporting the conclusions of this article will be made available by the authors, without undue reservation.

## Ethics statement

The studies involving humans were approved by the Ethics Review Board of the National Center for Health Statistics approved all NHANES protocols, and written informed consents were obtained from all participants or their proxies. The studies were conducted in accordance with the local legislation and institutional requirements. The participants provided their written informed consent to participate in this study.

## Author contributions

ND: Conceptualization, Data curation, Writing – original draft. ZZ: Formal Analysis, Methodology, Writing – original draft. JL: Investigation, Writing – original draft. KL: Conceptualization, Methodology, Software, Writing – review & editing.
