# Supplementary material for: The cross-sectional relationship between vitamin C and high-sensitivity C-reactive protein levels: insights from NHANES database
Source: Front Nutr. 2023 Nov 10;10:1290749. doi: 10.3389/fnut.2023.1290749 (PMC10675847; doi:10.3389/fnut.2023.1290749)
Supplement: Supplementary file 1 [file Table_1.docx]

**Table S1. Univariate Analysis for hs-CRP (mg/L)**

| **Covariate** | **Statistics** | **Effect Size (β)** | **P-value** |
| --- | --- | --- | --- |
| Vitamin C | 53.098 ± 28.888 | -0.034 (-0.041, -0.027) | <0.00001 |
| Vitamin C Categories |  |  |  |
| <11 | 323 (6.004%) | Reference |  |
| >=11, <24 | 623 (11.580%) | -0.766 (-1.750, 0.219) | 0.12752 |
| >=24, <50 | 1533 (28.494%) | -1.317 (-2.212, -0.422) | 0.00395 |
| >=50, <70 | 1538 (28.587%) | -2.762 (-3.651, -1.873) | <0.00001 |
| >=70 | 1363 (25.335%) | -3.032 (-3.935, -2.129) | <0.00001 |
| Age | 47.343 ± 21.171 | 0.026 (0.015, 0.036) | <0.00001 |
| DII | 1.612 ± 1.786 | 0.322(0.210, 0.434) | <0.00001 |
| Sex |  |  |  |
| Female | 2774 (51.561%) | Reference |  |
| Male | 2606 (48.439%) | -1.171 (-1.576, -0.766) | <0.00001 |
| Race/Ethnicity |  |  |  |
| Non-Hispanic Black | 1217 (22.621%) | Reference |  |
| Other Race - Including Multi-Racial | 1038 (19.294%) | -0.837 (-1.716, 0.043) | 0.06222 |
| Non-Hispanic White | 1853 (34.442%) | -0.766 (-1.434, -0.097) | 0.02478 |
| Mexican American | 764 (14.201%) | -0.446 (-1.349, 0.457) | 0.33314 |
| Other Hispanic | 508 (9.442%) | -1.100 (-2.083, -0.117) | 0.02839 |
| BMI | 28.760 ± 7.848 | 0.254 (0.228, 0.280) | <0.00001 |
| Supplement use |  |  |  |
| Yes | 2913 (54.145%) | Reference |  |
| No | 2462 (45.762%) | -0.376 (-0.786, 0.034) | 0.07202 |
| Not Recorded | 5 (0.093%) | -0.808 (-11.229, 9.612) | 0.87914 |
| Food Insecure |  |  |  |
| No | 3455 (64.219%) | Reference |  |
| Yes | 1678 (31.190%) | 1.398 (0.917, 1.878) | <0.00001 |
| Not Recorded | 247 (4.591%) | 0.154 (-0.916, 1.224) | 0.77762 |
| Physical activity |  |  |  |
| No | 602 (11.190%) | Reference |  |
| Yes | 2985 (55.483%) | -0.869 (-1.540, -0.197) | 0.01123 |
| Not Recorded | 1793 (33.327%) | -0.104 (-0.845, 0.636) | 0.78251 |
| Family PIR |  |  |  |
| <=1.5 | 1661 (30.874%) | Reference |  |
| >1.5, <=4.5 | 2027 (37.677%) | -0.510 (-1.054, 0.033) | 0.06557 |
| >4.5 | 1002 (18.625%) | -1.163 (-1.733, -0.593) | 0.00006 |
| Not Recorded | 690 (12.825%) | -0.212 (-0.974, 0.551) | 0.58612 |
| Alcohol consumption |  |  |  |
| No drinking | 452 (8.401%) | Reference |  |
| Drinking | 3122 (58.030%) | -0.408 (-1.246, 0.429) | 0.33921 |
| Not Recorded | 1806 (33.569%) | 0.259 (-0.639, 1.157) | 0.57143 |
| Smoking Status |  |  |  |
| Former | 1180 (21.933%) | Reference |  |
| Never | 2814 (52.305%) | -0.494 (-0.991, 0.002) | 0.05109 |
| Now | 870 (16.171%) | 0.030 (-0.619, 0.679) | 0.92765 |
| Not Recorded | 516 (9.591%) | -2.649 (-3.630, -1.667) | <0.00001 |
| Diabetes |  |  |  |
| No | 3911 (72.695%) | Reference |  |
| Borderline | 347 (6.450%) | 1.465 (0.688, 2.243) | 0.00022 |
| Yes | 1078 (20.037%) | 3.495 (2.936, 4.054) | <0.00001 |
| Not Recorded | 44 (0.818%) | 4.209 (2.096, 6.323) | 0.00010 |
| Hypertension |  |  |  |
| Yes | 2274 (42.268%) | Reference |  |
| No | 2862 (53.197%) | -1.517 (-1.935, -1.098) | <0.00001 |
| Not Recorded | 244 (4.535%) | -3.214 (-4.552, -1.876) | <0.00001 |

Abbreviations: CI, confidence interval; BMI, body mass index; PIR, household income-to-poverty ratio; hs-CRP, High Sensitivity C-reactive Protein; DII, dietary inflammatory index.
