# Supplementary material for: The cross-sectional relationship between vitamin C and high-sensitivity C-reactive protein levels: insights from NHANES database
Source: Front Nutr. 2023 Nov 10;10:1290749. doi: 10.3389/fnut.2023.1290749 (PMC10675847; doi:10.3389/fnut.2023.1290749)
Supplement: Supplementary file 2 [file Table_2.DOCX]

**Table S2 Effect size of vitamin C (µmol/L) on hs-CRP (mg/L) in prespecified and exploratory subgroups in the subgroup using supplements**

| Supplement Use | Yes  β (95%CI) *P*-value | No  β (95%CI) *P*-value | Not recorded  β (95%CI) *P*-value | *P* for interaction |
| --- | --- | --- | --- | --- |
| No. of participants | 2391 | 2462 | 5 |  |
| Crude | -0.042 (-0.052, -0.033) <0.0001 | -0.035 (-0.047, -0.023) <0.0001 | 0.007 (-0.510, 0.524) 0.9783 | 0.6253 |
| Model I | -0.046 (-0.056, -0.037) <0.0001 | -0.034 (-0.047, -0.022) <0.0001 | -0.025 (-0.539, 0.489) 0.9230 | 0.2968 |
| Model II | -0.028 (-0.037, -0.019) <0.0001 | -0.015(-0.028, -0.003) 0.0180 | 0.022 (-0.476, 0.519) 0.9317 | 0.2689 |

Abbreviations: CI, confidence interval; hs-CRP, High Sensitivity C-reactive Protein.

Crude Model no covariates were adjusted.

Model I adjusted for sex, age, and race/ethnicity.

Model II adjusted for sex, age, race/ethnicity, family poverty income ratio level, body mass index, hypertension, diabetes, physical activity, food insecure, body mass index, smoking status and alcohol consumption, dietary inflammatory index.
